# Endogenous Caulimovirids: Fossils, Zombies, and Living in Plant Genomes

**DOI:** 10.3390/biom13071069

**Published:** 2023-07-03

**Authors:** Héléna Vassilieff, Andrew D. W. Geering, Nathalie Choisne, Pierre-Yves Teycheney, Florian Maumus

**Affiliations:** 1 INRAE, URGI, Université Paris-Saclay, 78026 Versailles, France; helena.vassilieff@inrae.fr (H.V.); nathalie.choisne@inrae.fr (N.C.); 2 Queensland Alliance for Agriculture and Food Innovation, The University of Queensland, Brisbane, QLD 4072, Australia; a.geering@uq.edu.au; 3 CIRAD, UMR PVBMT, F-97410 Saint-Pierre de La Réunion, France; 4 UMR PVBMT, Université de la Réunion, F-97410 Saint-Pierre de La Réunion, France

**Keywords:** *Caulimoviridae*, pararetrovirus, endogenous viral elements, plant genomes, repetitive elements, centromeres, paleovirology

## Abstract

The *Caulimoviridae* is a family of double-stranded DNA viruses that infect plants. The genomes of most vascular plants contain endogenous caulimovirids (ECVs), a class of repetitive DNA elements that is abundant in some plant genomes, resulting from the integration of viral DNA in the chromosomes of germline cells during episodes of infection that have sometimes occurred millions of years ago. In this review, we reflect on 25 years of research on ECVs that has shown that members of the *Caulimoviridae* have occupied an unprecedented range of ecological niches over time and shed light on their diversity and macroevolution. We highlight gaps in knowledge and prospects of future research fueled by increased access to plant genome sequence data and new tools for genome annotation for addressing the extent, impact, and role of ECVs on plant biology and the origin and evolutionary trajectories of the *Caulimoviridae*.

## 1. Introduction

Endogenous viral elements (EVEs) are viral sequences that are embedded in host genomes and transmitted from one generation to the next like normal cellular genes. Vertical transmission of EVEs only occurs when the initial integration event takes place in the germline cells. Prophages and endogenous retroviruses (ERVs) were the first EVEs to be discovered in the 1950s and 1960s, respectively [1,2], and have been the subject of much research as ERVs in particular are responsible for important human diseases such as multiple sclerosis, rheumatoid arthritis, and cancer [3]. More recently, non-retroviral EVEs originating from viruses with single-stranded (ss) DNA, double-stranded (ds) DNA, ssRNA, or dsRNA genomes have been discovered in the genomes of a wide range of eukaryotic and prokaryotic organisms [4,5]. The first report of a plant EVE came in 1996 with the detection of geminivirus-related DNA (GRD) in the genome of tobacco (*Nicotiana tabacum*) [6,7]. This was followed by the discovery of DNA with homology to members of the *Caulimoviridae* in the genomes of tobacco and banana (*Musa balbisiana*) in 1999 [8,9,10]. The majority of characterized plant EVEs originate from the family *Caulimoviridae*, which contains viruses that have a circular, non-covalently closed dsDNA genome [11]. The *Caulimoviridae* is one of five families in the order *Ortervirales*, which also includes the *Retroviridae*, *Belpaoviridae* (LTR retrotransposons), *Metaviridae* (Ty3/Gypsy elements), and *Pseudoviridae* (Ty1/Copia elements) [12]. The feature that unites all members of *Ortervirales* is the presence of a *gag*-*pol* replication core unit. The *pol* (polymerase) gene encodes a polyprotein containing highly conserved aspartic protease, reverse transcriptase, and ribonuclease H1 enzymes, while the *gag* (group antigen) gene encodes proteins that are major components of the virion capsid. During replication, the viral genome alternates between dsDNA and ssRNA through cycles of transcription and reverse transcription. The genomes of viruses in the families *Retroviridae*, *Belpaoviridae*, *Metaviridae*, and *Pseudoviridae* encode a *pol* gene with an integrase domain, contrary to that of *Caulimoviridae*.

Historically, members of the *Hepadnaviridae*, a family of animal viruses with dsDNA genomes, and the *Caulimoviridae*, have been referred to as “pararetroviruses”, as in common with retroviruses, they replicate by reverse transcription. However, members of the *Caulimoviridae* and *Hepadnaviridae* differ from retroviruses by encapsidating dsDNA instead of ssRNA in the virion, and there is no proviral stage in the replication cycle since they do not encode an integrase. In this review, we adopt the name ‘endogenous caulimovirid (ECV)’ instead of ‘endogenous pararetrovirus’, as “pararetrovirus” is a descriptive term with no taxonomic meaning [12].

**Figure 1:**
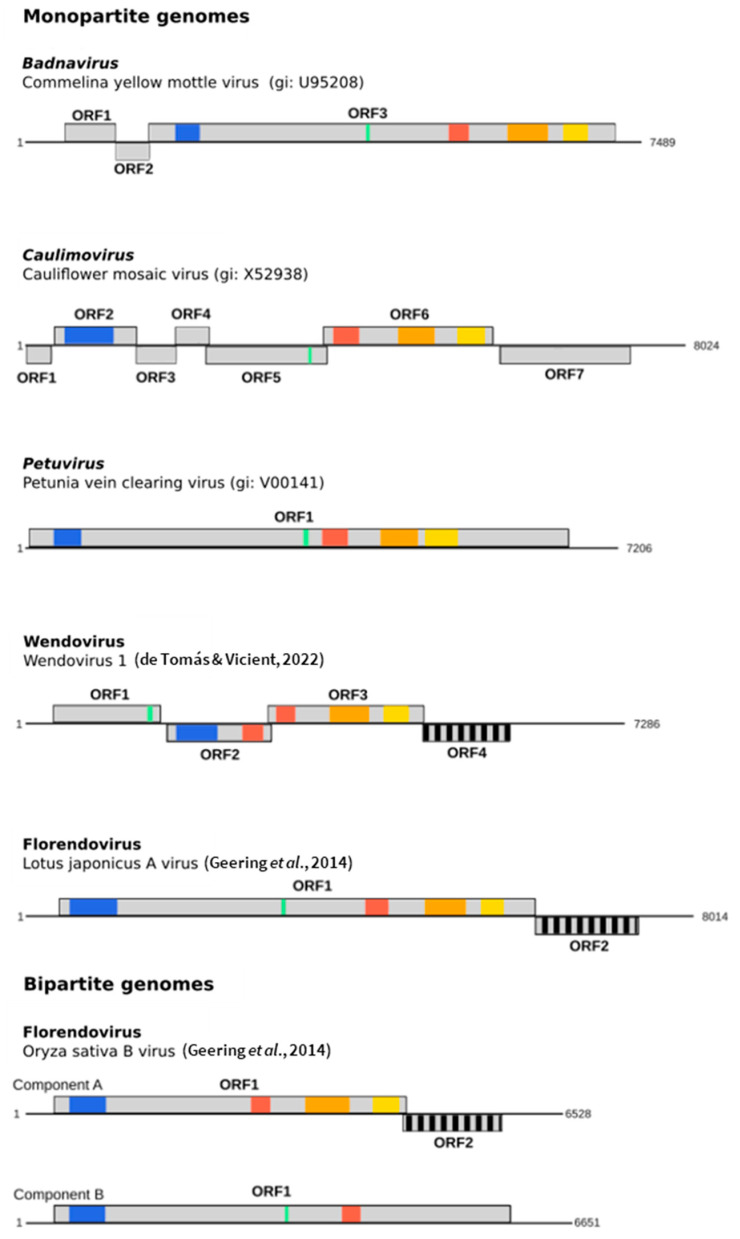
Genomic organization of episomal and endogenous *Caulimoviridae*. Schematic representation of the genomes of petunia vein-clearing virus (PVCV, type member of genus *Petuvirus*), Commelina yellow mottle virus (ComYMV, type member of genus *Badnavirus*), cauliflower mosaic virus (CaMV, type member of genus *Caulimovirus*), Wendovirus 1 (Wendo1, type member of tentative genus Wendovirus) [16], Lotus japonicus A virus (LjapAV, member of tentative genus florendovirus) [17], and Oryza sativa B virus components A and B (OsatBV compA&B, a florendovirus with a bipartite genome) [17]. Light gray boxes mark open reading frames, and colored regions within ORFs are conserved protein domains: blue is the viral MP domain; green is a zinc-finger domain within the coat protein; red is the retropepsin (pepsin-like AP) domain; orange is the reverse transcriptase domain; and yellow is the RNaseH1 domain. ORFs with unknown functions are cross-hatched. Genbank accession numbers of type members of the viral genera are provided. ORFs were predicted using ORFinder (https://www.ncbi.nlm.nih.gov/orffinder/, accessed on 1 March 2023), and conserved domains were predicted using MOTIF search (https://www.genome.jp/tools/motif/, accessed on 1 March 2023) with Pfam and CDD database.

The International Committee on Taxonomy of Viruses (ICTV) currently recognizes 11 genera in the *Caulimoviridae* based on virion shape, genome organization, vector group, and minor variations in the replication cycle. These genera are *Badnavirus*, *Caulimovirus*, *Cavemovirus*, *Dioscovirus*, *Petuvirus*, *Rosadnavirus*, *Ruflodivirus*, *Solendovirus*, *Soymovirus*, *Tungrovirus*, and *Vaccinivirus* [13]. The genomes of members of the *Caulimoviridae* range in size between 7.1 and 9.8 kbp and contain one to nine open reading frames (ORFs) (Figure 1). Caulimovirid proteomes invariably comprise a movement protein (MP), a capsid protein (CP), and a polymerase polyprotein containing aspartic protease (AP) and reverse transcriptase (RT) proteins, with an RNAse H1 (RH1) domain tethered to the RT protein [14,15]. Auxiliary proteins that are limited to one or a subset of genera are also produced, such as the aphid transmission factor in the case of caulimo- and soymoviruses [13].

Over the last 10 years, there have been a growing number of studies on ECVs, aided by exponential growth in the number of plant genome sequence resources. This review aims at providing an overview of the current knowledge of the diversity, host range, abundance, and genomic distribution of ECVs. We also discuss their putative functions, integration modes, and the way they inform the long-term evolution of the *Caulimoviridae*.

## 2. ECVs as Molecular Fossils of *Caulimoviridae*

### 2.1. Classification of ECVs

ECVs are relics of past infections that occurred thousands to millions of years ago (mya) [5] and therefore are often referred to as ‘molecular fossils’. Upon integration of a viral sequence in a plant genome, the selection pressures on this sequence dramatically change; the rate of mutation slows down and in the absence of autonomous viral replication, mutations in open reading frames that would previously have rendered the virus inactive are no longer eliminated through selection [4]. It is generally assumed that the mutations that do accumulate are random in distribution. Hence, the sequence of the viral genome at the time of integration can be estimated by aligning closely related EVE sequences and obtaining the consensus sequence. Following this basic principle, many entire viral genome sequences have been reconstructed from EVEs, allowing assignment of the viruses to either known or novel taxa [5]. A large amount of information can be gleaned from these reconstructed ancestral viral genome sequences such as genome architecture and even minute details about virus replication such as ribosome shunting during protein translation [18].

More than 100 complete caulimovirid genomes have been assembled from EVEs since the first ECVs were discovered in 1999. Efforts have been made to classify these sequences in the same way that fossils are recognized in the Linnaean taxonomy of all kingdoms of living organisms [19]. It should be noted that taxa are artificial concepts and that ECVs are not viruses *per se* but provide strong evidence of the existence of viruses that once existed.

Theoretical and practical impediments exist that currently prevent the recognition of ancestral viruses in the taxonomic framework advocated by the ICTV. Firstly, no matter how accurate the reconstruction of an ancestral viral genome sequence is, the replication of these sequences has not been experimentally verified. However, the same is true for the vast majority of new virus species that are recognized purely on the basis of a novel genome sequence generated by high throughput sequencing without supporting biological information such as evidence of infectivity, virion morphology, mode of transmission, and sometimes even knowledge about the host organism in the case of viruses discovered in environmental samples [20,21]. Secondly, for a virus species or genus to be formally recognized, a unique GenBank accession has to be mandatorily provided in the TaxoProp database. A scaffold of the host genome with nucleotide coordinates of the element is not sufficient. This problem has been overcome for members of the *Metaviridae* by depositing sequences in the Third-Party Annotation database of the International Nucleotide Sequence Database Collaboration (M. Krupovic, pers. comm.)

Two alternative naming conventions have been suggested for ECVs. Staginnus et al., 2009 recommended that when a cognate exogenous virus is not known to exist, then the EVEs should simply be labeled using the host plant initials followed by the suffix ‘EPRS’, whereby EPRS stands for endogenous pararetrovirus sequence. However, this naming convention fails to satisfy two of the most important objectives of a classification scheme: (i) describe how the various EVEs are related to each other and to exogenous viruses, and (ii) provide an ability to predict the essential features of the EVE based on referral to the name. To address these issues, Geering et al., 2010 adopted the premise that EVEs were molecular fossils of viruses that once existed and classified EVEs into tentative genera based on criteria such as genome organization and relatedness of the RT gene sequences. Following these criteria, two new viral genera were proposed, Orendovirus and Solendovirus [22]. By proposing the name ‘Orendovirus’, confusion was avoided that rice tungro bacilliform virus-like (eRTBVs) sequences are integrated in the rice genome, as initially proposed by Kunii et al. [23]. Although the eRTBVs were indisputably derivatives of viruses in the family *Caulimoviridae*, the ancestral viruses of these elements are so distantly related to rice tungro bacilliform virus as to constitute a new genus.

The diversity of ECVs known to occur has greatly expanded as more and more plant genomes and transcriptomes have become available. In 2014, Geering et al. [17] screened 32 angiosperm genomes for ECVs and manually assembled 76 entire or nearly full-length viral genomes from these EVEs, representing 34 distinct caulimovirid species based on an 80% nucleotide identity demarcation threshold in the RT-RNase H1 coding region [11]. Examination of genome organization combined with a phylogenetic analysis provided a strong basis to propose a new genus within the family *Caulimoviridae*, named florendovirus, after Flora, the Roman goddess of flowers, and ‘endovirus’, a contraction of endogenous virus (Flora endogenous virus).

In a second major study, Diop et al., 2018 examined a wider range of vascular plants (tracheophytes) [11]. Sequences of interest were first conceptually translated and then clustered using a 55% amino acid identity demarcation threshold for the RT domain. This simple step allowed the RT sequences from ICTV-approved genera to be sorted into distinct clusters, although cavemo- and solendoviral-like sequences could not be separated. This clustering enabled discovery of three novel operational taxonomic units (OTUs) in angiosperms that were named xendo-, yendo-, and zendovirus, respectively. An additional three groups containing a single viral species were similarly named after their respective host plant species (petunia-, glycine-, and vitis-endovirus, respectively). In addition, the work of Diop et al. [11] revealed the existence of four distinct OTUs in gymnosperms and two in ferns, that were named Gymnendovirus 1 to 4 and Fernendovirus 1 and 2, respectively.

In a concomitant study, Gong and Han [24] characterized two novel OTUs in ferns, named α- and β-fern endogenous caulimovirus-like (FEVC), and five in gymnosperms, named α-, β-, Ɣ-, δ-, and ε-gymnosperm endogenous caulimovirus-like (GECV). To reconcile the OTUs identified in gymnosperms by Diop et al. and Gong and Han, we clustered the RT sequences of their type members using a 55% identity cutoff (Table 1). Our work shows that four out of the five GECV from Gong and Han (GECV β, c, δ, ε) share similarities above the cutoff value with all of the four gymnendovirus genera from Diop et al. [11] (gymnendovirus 1, 2, 3, 4), respectively.

The list of novel ECVs continues to expand. A putative new genus named ‘Wendovirus’ was proposed by Tomas and Vicient [16] based on ECVs identified in the genomes of 11 angiosperms [12]. A distinctive feature of the genome organization of these viral sequences was the presence of two distinct aspartic protease domains (Figure 1) [16].

### 2.2. Caulimovirids Have Colonized Almost All Plant Families

Most extant members of the *Caulimoviridae* have narrow host ranges, restricted to species in one or a small number of plant families [13]. This observation may simply reflect a lack of field survey efforts or, alternatively, barriers to transmission through vector feeding preferences, as experimental host ranges determined using techniques such as agroinoculation are larger than those observed in nature [25,26]. The examination of ECVs has affirmed the notion that individual virus species in the *Caulimoviridae* have narrow host ranges, as distinct ECVs are typically confined to a single host species. Only endogenous tobacco vein-clearing virus has been detected in more than one plant species, these being South American representatives of *Solanaceae*, including a human-created *Nicotiana* hybrid (*Nicotiana* × *edwardsonii*) and *Solanum lycopersicum* [20,27].

What has become apparent from the study of ECVs is that members of *Caulimoviridae* have historically occupied a far greater range of ecological niches than is currently observed. In a survey of 66 tracheophyte genomes (four gymnosperms and 62 angiosperms) by Diop et al., 2018, at least one unique caulimovirid RT domain was observed in every examined plant species bar six, namely *Zea mays*, *Zostera marina*, *Oryza brachyantha*, *Arabidopsis thaliana*, *Schrenkiella parvula*, and *Carica papaya*. Remarkably, ECVs have been detected in virtually all major groups of higher plants, including ferns, gymnosperms, ANITA-grade angiosperms, magnoliids, monocots, and eudicots [6,11,12]). Beyond euphyllophytes, a transcript of a caulimovirid sequence was identified in a clubmoss (*Lycopodium annotinum*) [11]. However, the source dataset was later reported to contain some contaminated samples (http://gigadb.org/dataset/100910, accessed on 1 March 2023); hence, this result needs to be substantiated.

Overall, ECVs have been reported in all tracheophyte divisions but not in chlorophytes nor in more basal clades of streptophytes, for which only a limited number of genome and transcriptome sequences are available. Several factors may explain why members of the *Caulimoviridae* are limited to vascular plants. Plant organs of very primitive plants differ markedly from those of the tracheophytes, especially by the absence of a vascular system, which plays a key role in the infection process [28]. Tracheophytes and more basal lineages also differ in the structure of their plasmodesmata [29], which are essential to the long-distance transport of viral RNAs bound to viral movement proteins such as those encoded by members of *Caulimoviridae* [30]. These differences may result in plants from basal lineages being unable to support infection by members of the *Caulimoviridae*.

### 2.3. ECVs Provide Insights into Caulimovirid Genome Evolution

A library of curated consensus sequences of florendoviral genomes was created by Geering et al. [22], allowing close examination of the genome architecture of this novel genus. Most florendoviruses have a conventional monopartite viral genome containing two ORFs: ORF1 encodes a polyprotein with the conserved CP, MP, AP, RT, and RH1 domains, whereas ORF2 encodes a protein of unknown function that lacks detectable homology to any caulimovirid protein (Figure 1). However, in several plant species including *Vitis vinifera*, *Oryza sativa*, and *Sorghum bicolor*, evidence for bipartite florendoviral genomes was obtained, whose components, labeled A and B, were predicted to carry complementary and partially redundant ORFs that together constituted a complete florendoviral genome (Figure 1). Additional evidence for florendoviruses with bipartite genomes was also reported from the analysis of EVEs found in the genome of sugar beet (*Beta vulgaris*) [31]. Interestingly, even though the putative bipartite florendoviruses have similar genome organizations, they do not share a most recent common ancestor, suggesting that these viruses have evolved on multiple independent occasions. For one bipartite virus, Vitis vinifera B virus, a potential non-segmented parental form of the virus was identified, Vitis vinifera A virus, illustrating the progression of partitioning of the viral genome. There are many examples of multipartite viral genomes but not among members of *Ortervirales*. Many hypotheses have been proposed as to the advantages and disadvantages of partitioning of the viral genome [32,33] but few are supported by empirical evidence.

### 2.4. Dating the Integration of ECVs

The first attempt to estimate the date of integration of ECVs was undertaken in 2008, using endogenous banana streak viruses (eBSVs) present in the genome of *Musa balbisiana* accession Pisang Klutuk Wulung (PKW) [34,35]. Two allelic copies of endogenous banana streak GF virus (eBSGFV) are flanked by a Ty3/Gypsy retrotransposon. Gayral et al. [34] hypothesized that integration of BSGFV DNA was mediated by the Ty3/Gypsy retrotransposon: recombination first occurred between the two retroelements and the resultant chimeric molecule inserted in the chromosome using the normal integration mechanism of the Ty3/Gypsy retrotransposon. Gayral et al. [34] dated the age of long terminal repeat (LTR) sequences associated with the Ty3/Gypsy LTR retrotransposon, as LTRs are identical upon insertion, but once the retrotransposon loses mobility, they accumulate mutations at the host neutral rate of evolution. Applying an average synonymous substitution rate for *Musaceae*, the authors inferred that the retrotransposon-BSGVF chimeric molecule integrated into the genome of PKW 640,000 years ago.

There are potential flaws in the dating approach taken by Gayral et al. [34]. Firstly, it is assumed that the integration of BSGFV and the Ty3/Gypsy retrotransposon occurred simultaneously, whereas it could have been a two-step process [34]. Furthermore, quantifying mutations in LTR pairs can sometimes be misleading owing to gene conversion events and saturation effects [36]. In addition, molecular clocks are not always well calibrated and can be unreliable when applied to very ancient sequences such as ERVs or other EVEs.

A second more reliable approach for dating the integration of EVEs relies on the identification of orthologous integration loci in different host species for which a dated phylogeny is available, preferably calibrated using the fossil record. Such loci can be detected either because integration occurred in a specific gene (e.g., within an intron), in syntenic positions (i.e., between the same two consecutive genes), or because these loci share identical flanking sequences. Orthologous EVEs are supposed to result from a single integration event that occurred at least in the last common ancestor of the host species sharing this homoplasy. For instance, the oldest known orthologous ERV is an ERV-L sequence that is found in the genome of placental mammals that diverged 104–110 million years ago (mya) [37]. Likewise, several orthologous ECVs were identified in plant genomes.

Geering et al. [11] found one integration locus of a partial genome sequence of Oryza sativa B virus (OsatBV), a florendovirus, in seven *Oryza* species. They inferred that the integration of this sequence occurred in the last common ancestor of these species, from which they diverged 1.8 mya, resulting in an estimated minimal age of OsatBV integration of 1.8 million years (myr) [22]. Gong and Han [23] similarly identified an orthologous integration locus in the genomes of *Picea glauca* and *Picea abies*, which diverged from a common ancestor 16.9 mya, and another one in the genomes of *Pinus taeda* and *Pinus lambertiana*, which diverged from a common ancestor 75 mya. Thus, the authors assumed that the minimum ages of the integration of the cognate viruses were 16.9 myr and 75 myr, respectively.

Lastly, the age of a viral genus can be estimated using biogeographical information, but this only dates to the age of the most recent common ancestor of the viral taxa and not the integration events. For example, there is a well-supported clade of florendoviruses from *Eucalyptus grandis*, an Australian plant, and *Gossypium raimondii* and *Theobroma cacao*, both South American plants [22]. The Drake Passage formed about 34 million years ago, severing the land bridge between South America, Antarctica, and ultimately Australia. This clade of florendoviruses could therefore be estimated to be at least this old. It is unlikely that florendoviruses naturally dispersed across the vast expanse of the Pacific Ocean.

### 2.5. Using ECVs to Infer the Long-Term Evolution of the Caulimoviridae

By providing access to ancient or hitherto unknown viral genome sequences, EVEs have the potential to help refine the evolutionary history of cognate viruses and corresponding viral genera and families. For example, foamy viruses (FVs) are a group of retroviruses in the genus *Spumavirus* with an exceptionally stable co-speciation pattern across eutherian mammals over at least 100 myr [38]. By evaluating the co-speciation between FVs, FV-like ERVs (FLERVs), and their hosts, Aiewaskun and Katzourakis proposed a macroevolution scenario in which viruses in the *Retroviridae* emerged in a marine vertebrate host of at least 460 mya and then co-evolved with their hosts with occasional host jumps [39].

**Figure 2:**
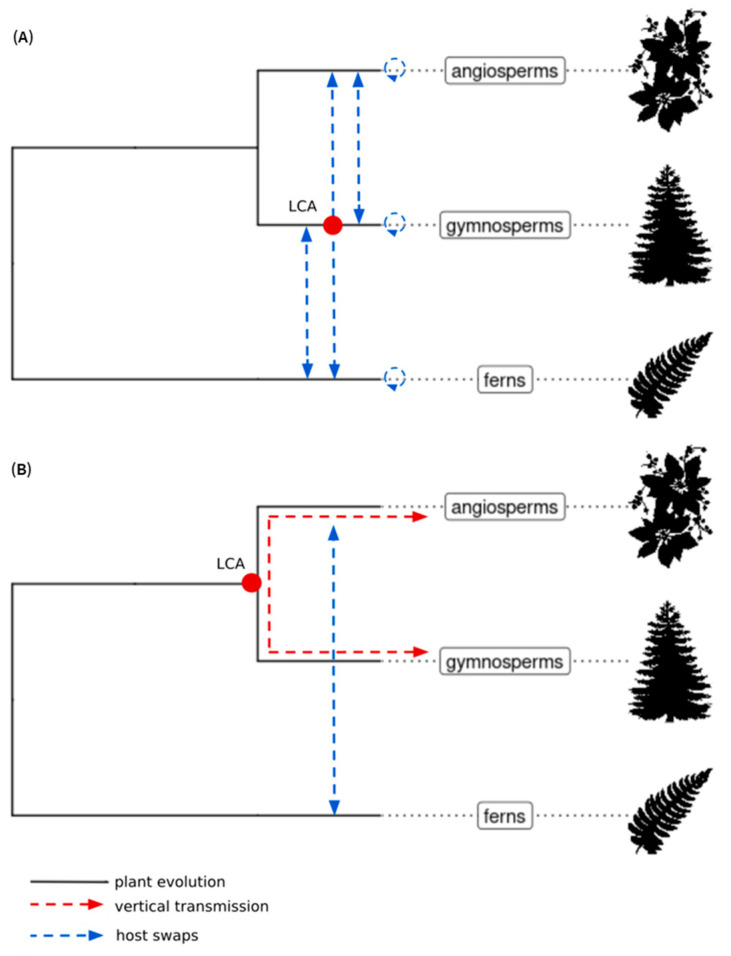
Two current scenarios of the macroevolution of *Caulimoviridae* in euphyllophytes.

Similar phylogenetic analyses performed on sequences of both ECVs and extant members of the *Caulimoviridae* provide insights into the macroevolution of *Caulimoviridae* over extended timeframes [11,23]. Using Bayesian phylogenetic reconstruction, Gong and Han [23] proposed that the *Caulimoviridae* originated either following coevolution with their euphyllophyte hosts over a 400 myr period with occasional cross-species transmission or through frequent and predominant cross-species transmission (Figure 2A).

**Figure 3:**
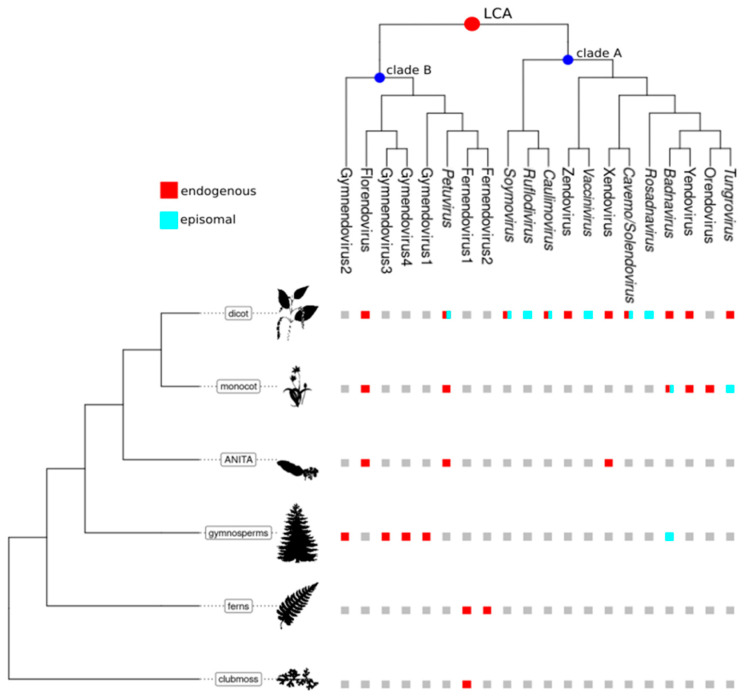
Distribution of endogenous and episomal *Caulimoviridae* among tracheophytes.

In a concomitant study, Diop et al. [11] analyzed the diversity of ECVs in the genomes of a wide range of angiosperms, a few gymnosperms and ferns. The gymnosperm and fern genomic data that they used were partially redundant with those used by Gong and Han [23], resulting in equally partially redundant sets of novel caulimovirid sequences in both studies (Table 1). Using maximum-likelihood phylogenetic analysis, Diop et al. identified two distinct monophyletic clades called A and B [11] (Figure 3). Clade A exclusively comprised sequences from endogenous and episomal members of the *Caulimoviridae* found in angiosperms, whereas clade B comprised sequences of ECVs from different tracheophyte divisions. Diop et al. [11] proposed that the *Caulimoviridae* emerged in a common ancestor of angiosperms and gymnosperms, which diverged 320 mya. Following cross division swaps, viruses in clade B would have infected angiosperms, leading to petuviruses and florendoviruses, and ferns, leading to fernendoviruses (Figure 2B).

Both evolutionary scenarios proposed by Gong and Han [23] and by Diop et al. [11] suppose an ancient origin of the *Caulimoviridae* (Figure 2). Some viral clades were only found in one or the other of the two studies, whereas other clades appear to be similar in both studies (Table 1). However, despite these similarities, the topologies of the phylogenetic trees obtained by Gong and Han and Diop et al., respectively, differ significantly. This difference may be attributable to differences in the type of phylogenetic analysis each group employed. For the time being, there is no reason to favor one macroevolutionary scenario or tree topology over the other. Further work involving a larger number of tracheophyte genomes, including basal ones, and using high-quality genome assemblies, will make it possible to refute, confirm, or complete these existing scenarios.

Macroevolution of *Caulimoviridae* according to Diop et al. [11] (A) and Gong and Han [23] (B; modified from Gong and Han, 2018). Cladograms indicate the main divisions of euphyllophytes. The position of the hypothetical last common ancestor (LCA) of *Caulimoviridae* is shown by a red dot. The red and blue arrows represent vertical transmission and host swaps, respectively. Graphic representations of plants were retrieved from the Phylopic database (https://www.phylopic.org/, accessed on 1 March 2023).

The left cladogram represents the evolutionary relationships between major classes of vascular plants. The upper cladogram represents the phylogeny of the *Caulimoviridae* based on the work of Diop et al. [11]. Red and blue dots show the position of the hypothetical last common ancestor (LCA) of the *Caulimoviridae* and that of clades A and B on the phylogenetic tree, respectively, according to Diop et al. [11]. At the intersection between both cladograms, colored boxes indicate the presence of endogenous (red) or episomal (blue) representatives of the *Caulimoviridae*. Graphic representations of plants were retrieved from the Phylopic database (https://www.phylopic.org/, accessed on 1 March 2023).

### 2.6. Origin of the Caulimoviridae

Phylogenetic analyses of the *Ortervirales* consistently support the monophyly of the *Caulimoviridae* and its placement as a sister clade to the *Metaviridae* [12,40,41,42]. At least four hypotheses can be proposed to explain this situation: (1) phylogenetic reconstruction is not accurate; (2) the *Caulimoviridae* emerged from the *Metaviridae* and represents an ancient lineage of this virus family; (3) the *Metaviridae* emerged from the *Caulimoviridae* and represents an ancient lineage of this family; (4) the *Caulimoviridae* and *Metaviridae* split from a most recent common ancestor.

The analysis of phylogenetic relationships within the order *Ortervirales* relies on the alignment of short (*c*. 300 aa for RT and 500 aa for RT-RNase H1) and poorly similar sequences, making the assignment of positional homology difficult, hence producing a weak estimation of phylogenetic relationships [43]. In addition, very different evolution rates apply within the order *Ortervirales* between endogenous elements (*Metaviridae* and EVEs), which are mostly replicated by the host machinery and subject to the host’s evolution rate, and episomal forms of viral genomes, which are replicated by error-prone RTs. Therefore, it cannot be ruled out that the observed sisterhood relationship between the *Caulimoviridae* and *Metaviridae* reflects an artefact of phylogenetic analyses (Hypothesis 1). An alternative approach, based on the comparison of a lower number of short and highly constrained nucleotide and protein sequences, resulted in an accurate estimate of the evolutionary relationships of retroviruses [44,45] and could be applied to the *Caulimoviridae* and *Metaviridae* to unravel their phylogenetic relationships.

**Figure 4:**
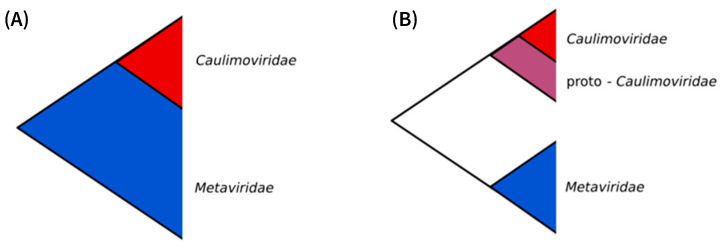
Two scenarios of the deep evolution of *Caulimoviridae* and *Metaviridae.* Hypothesis 2 (**A**): the *Caulimoviridae* emerged from the *Metaviridae* and represents an ancient lineage of the *Metaviridae*; Hypothesis 4 (**B**): the *Caulimoviridae* and *Metaviridae* evolved independently from a common ancestor.

The *Metaviridae* forms a monophyletic group that is ubiquitous across all eukaryotic supergroups, and their phylogeny largely mimics that of their eukaryotic hosts [46], suggesting that this family existed at the time of the last eukaryotic common ancestor (LECA), i.e., at least 1.2 billion years ago [47]. The *Caulimoviridae* is thought to have emerged more recently. All known members of the *Caulimoviridae* (episomal and endogenous alike) share a monophyletic MP that probably was acquired from a ssRNA virus [28,48]. Taking the acquisition of an MP as a founding event that promoted the spread of the *Caulimoviridae* to all subdivisions of the tracheophytes [11], the maximum age for the emergence of the *Caulimoviridae* can be considered contemporaneous with the emergence of vascular plants, i.e., about 420 mya [49,50]. If the *Caulimoviridae* did emerge from the *Metaviridae* (Hypothesis 2), one would expect phylogenetic analyses to place the *Caulimoviridae* as a nested clade within the *Metaviridae*, which is not the case. However, Hypothesis 2 could stand if the observed sisterhood relationship reflects incomplete lineage sorting since the *Caulimoviridae* could have derived from a yet unknown lineage of the *Metaviridae* (Figure 4A).

The probable late emergence of the *Caulimoviridae* after the *Metaviridae* is also against Hypothesis 3. In addition, the *Metaviridae* shares more plesiomorphic traits with other families in the order *Ortervirales* than with *Caulimoviridae*, which lack integrase and LTRs [12]. This further suggests that the *Metaviridae* is unlikely to derive from *Caulimoviridae*.

Alternatively (Hypothesis 4), the *Caulimoviridae* could have evolved from ancient *Ortervirales* (referred to as proto-*Caulimoviridae*), sharing a common ancestor with *Metaviridae* after they acquired the MP gene (Figure 4B). This scenario either assumes that *Caulimoviridae* are the only modern representatives of this ancient *Ortervirales* lineage and/or that our knowledge of the diversity of modern and ancient *Ortervirales* is only partial.

## 3. Integration, Genomic Distribution, and Expression of ECVs

### 3.1. Mechanisms of Integration

The mechanism(s) by which caulimovirid DNA is captured in plant genomes remain(s) speculative. However, there are two widely held hypotheses, the first implicating repair mechanisms for dsDNA breaks in chromosomes and the second involving the integration machinery of LTR retrotransposons.

ECV loci are often flanked by TA dinucleotide simple repeats ((TA)n) [21,22,31,51,52]. These simple repeats are palindromic in nature and can form stable non-B DNA structures (e.g., cruciform and hairpin structures) that stall the replication fork and lead to double-strand breaks (DSBs) in the chromosomes through local fragility or from the action of DNA nucleases [53]. The integration bias of ECVs toward (TA)n repeats may thus result from the insertion of caulimovirid DNA as filler DNA during the repair of these DSBs, by non-homologous end joining (NHEJ) or microhomology-mediated recombination [54].

ECVs have been frequently observed to form tandem arrays and multicopy rearrangements [22,31,55,56,57], sometimes leading to integration hotspots (see below). Such structures could arise directly from the integration of concatemers of viral DNA or from homologous recombination either between existing ECVs or between an ECV and the episomal form of a cognate virus. Interestingly, a recent study has demonstrated the capability of the fission yeast Tf1 LTR retrotransposon to transpose through an integrase-independent pathway. In this system, Tf1 transposition occurs by homologous recombination mediated by the single-strand annealing protein Rad52 and not by canonical homologous recombination that would require Rad51, resulting predominantly in insertions within existing copies of related elements [58]. Rad52 homologs were identified in gymnosperms, monocots, and dicots [59] and could promote the integration of ECVs by a similar homologous recombination mechanism.

An alternative path for the integration of ECVs could involve retrotransposons through recombination between a caulimovirid genome and an LTR retrotransposon, followed by the integration of the resulting chimera. Such a mechanism has been suggested for the integration of badnaviruses in the genome of *Musa balbisiana* [34] and that of bornaviruses [60] and flaviviruses [61] in the genomes of mammals and mosquitoes, respectively. It has also been suggested that caulimovirid integration could involve the hijacking of a transposon-derived integrase [62].

### 3.2. How Caulimovirid EVEs Have Invaded Plant Genomes

The tendency of ECVs to integrate into hotspots and their abundance raises the question of their accumulation and maintenance in plant genomes. In principle, each ECV results from a single integration event, except those resulting from segmental duplications, whole genome duplications, or natural hybridization. In many plant genomes, there is evidence of recent integration events, reflected by the presence of highly similar copies of ECVs. For example, de Tomas and Vicient [24] reported over 30 clusters of identical ECVs in angiosperm genomes spanning different plant genera, suggestive of very recent integration events. Most of these clusters correspond to petuviruses and florendoviruses for which no episomal form has been reported. At least two hypotheses could explain this situation: either (i) these viruses became extinct recently, or (ii) they are still extant and cause asymptomatic or unnoticed infection; hence, they were never isolated. The first hypothesis would involve massive virus extinction events, which cannot be ruled out *a priori*. Although most extant members of the *Caulimoviridae* cause disease symptoms, asymptomatic viral infections are produced by some badnaviruses such as taro bacilliform virus and are commonplace in plants; therefore, the second hypothesis cannot be ruled out either [63].

Alternatively, repetitive ECV could result from endogenous proliferation, which would not require an episomal stage: viral dsDNA would rather be produced from replication-competent EVEs or in *trans* by complementation of defective EVEs by episomal counterparts. Such a situation exists for the endogenous retrovirus group HERV-K (HML2) in primates, for which proliferation likely results from the reinfection of germline cells [64]. In plants, genes encoding caulimovirid MP homologs are widespread in the genomes of euphyllophytes [28]. Their constitutive expression could facilitate cell-to-cell movement of caulimovirid virions and promote iterative infection of plant reproductive tissues, resulting in repetitive ECVs.

### 3.3. Distribution of Caulimovirid EVEs in Plant Genomes

Only a few studies have attempted to quantify the contribution of ECVs to angiosperm genomes, one being that of Kim et al. [65], who estimated that caulimovirid sequences represent 0.86% and 1.02% of the *Capsicum annuum* and *Capsicum chinensis* genome assemblies, respectively. The abundance of ECVs in plant genomes is very variable, ranging from trace amounts to above 0.5% of the genome content for *Jatropha curcas*, *Amborella trichopoda*, *Citrus clementina*, *Vitis vinifera*, and *Beta vulgaris*, and above 1% in *Ricinus communis* and *Solanum melongena* [22,31,66].

To provide a rough estimate of the relative abundance of ECVs across tracheophytes, Diop et al. [11] counted the number of ECV reverse transcriptase (ECRT) sequences in various plant genomes and normalized this data according to the size of the plant genome. With a few exceptions, they found ECRTs in all the genomes of the ferns, gymnosperms, and angiosperms they analyzed, albeit at highly variable densities. Overall, ECRTs were relatively abundant in gymnosperms (over 500 copies per genome) and less abundant in monocots than in dicots, among which some botanic orders (e.g., *Solanales* and *Malpighiales*) tend to display high ECRT densities. The highest densities were registered in *Citrus sinensis* (sweet orange) and *Ricinus communis* (castor bean) at 2.3 and 2 ECRTs per Mb, respectively, compared to an average density of 0.2 ECRT/Mb across the 62 seed plant species that were analyzed [11].

The distribution of ECVs along host chromosomes has been investigated in several ECRT-rich angiosperm genomes. In silico and cytological analysis showed that ECVs are present in all chromosomes of *Citrus maxima* [52], *Medicago truncatula* [67], *Beta vulgaris* [31], *Solanum lycopersicum* [68], *Petunia hybrida* [55], and *Fritillaria imperialis* L. [69]. In the genome of *Capsicum annuum*, which contains about 1,500 ECRTs, their distribution was found to be homogeneous along all chromosomes [65]. However, ECVs often tend to cluster in hotspots, which can be unevenly distributed across chromosomes [31,52,55,67,68,69,70]. For example, hotspots of petuvirus EVEs were observed in most, if not all, centromeres of the giant genome of monocotyledonous species *Fritillaria imperialis*, whose estimated size is ~42 Gbp [69]. This situation could result from a recent host swap since the genome of closely related *Fritillaria* species is devoid of petuvirus EVEs [69]. ECVs were also found to be a component of centromeric repeats in citrus hybrids [71], suggesting that they could play a role in the centromeres of different plant species.

In a majority of plant genomes, ECVs were found to be frequently located in close proximity to transposable elements (TEs) [22,52]. In the genomes of *Beta vulgaris*, *Petunia hybrida*, and *Solanum lycopersicum*, they localize preferentially in gene-poor, TE-rich pericentromeric regions [31,55,68]. This preferential distribution could result from genome-wide selection against deleterious events, such as EVE integrations, which are more likely to occur in gene-rich regions where integrants have the potential to impact both the structure and expression pathways of host genes [66]. Therefore, as for TEs [72,73], heterochromatin/pericentromeric regions may represent “safe havens” for ECVs, where they can escape purge mechanisms and their retention is neutral to the host and can last over extended evolutionary periods.

### 3.4. Replication-Competent ECVs

Replication-competent ECVs have been reported only for endogenous banana streak viruses (eBSVs) in banana, endogenous petunia vein-clearing virus (ePVCV) in petunia, and endogenous tobacco vein-clearing virus (eTVCV) in *Nicotiana edwardsonii* [27,55,57]. They are the only currently known examples of infective EVEs in plants, although it is likely that others will be discovered. Activation by biotic and abiotic stresses triggers their expression, leading to spontaneous infections; therefore, they are often referred to as replication-competent EVEs. Replication-competent eBSVs were discovered in 1999 [7,8], but their complex structure was elucidated only in 2013 [57]. Chabannes et al. [57] discovered that infective eBSVs of three BSV species, BSOLV, BSGFV, and BSIMV, are present as allelic forms in *Musa balbisiana* genomes: eBSOLVs and eBSGFVs are di-allelic, and only one allele is infective, whereas eBSIMV is mono-allelic, although there are minor sequence differences between alleles, and both alleles are infective [57]. The structure and/or infective nature of eTVCVs and ePVCVs were unraveled in 2000 [27] and 2003 [55], respectively. The mechanisms of activation of infective ECVs are still poorly understood; however, wounding, temperature differences, water stress, grafting, and cell culture have been identified as activating stresses [55,74,75,76], and activation has been observed only in interspecific hybrids. *Nicotiana edwardsonii* is a hybrid between *Nicotiana glutinosa*, whose genome harbors infective eTVCVs, and *Nicotiana clevelandii* [27]. Likewise, *Petunia hybrida* results from a cross between *Petunia inflata* and *Petunia axillaris* subsp. *axillaris* [37], whose genomes both harbor infective ePVCVs. Finally, activation of infective eBSVs occurs in triploid (AAB) or tetraploid (AAAB) interspecific banana hybrids resulting from crosses between *Musa balbisiana* (B genome), which harbors infectious eBSVS, and *Musa acuminata* (A genome) [57].

Two mechanisms have been proposed to explain the release of episomal viral genomes from ECVs. They depend on the structure and location of the ECVs in the host genome. Endogenous PVCVs are integrated into tandem arrays in the genome of *Petunia hybrida*. They form head-to-tail concatemers, allowing transcription from the promoter of the first copy to the polyadenylation site of the next, resulting in transcripts of complete viral genomes that would be infectious [55]. In *Musa balbisiana*, replication-competent eBSVs display complex rearrangements. Fragmented viral sequences are often duplicated in the same or opposite directions [57]. A homologous recombination (HR)-based model has been proposed to explain the release of a banana streak GF virus (BSGFV) genome from its endogenous counterpart, eBSGFV-7, which requires two recombination events [57]. The first one between two inverted repeat sequences would produce a complete linear copy of the infectious eBSV with its three ORFs in the same orientation. The second one would excise a viral circular molecule resembling the cognate BSV genome, from which infection could arise. It is important to note that the insertion of infective ePVCVs and eBSVs is supposed to be relatively recent, preventing the accumulation of mutations and sequence decay that generally make EVEs nonfunctional [55,57].

### 3.5. Epigenetics and Silencing

The epigenetic status of ECVs has only been addressed in a few angiosperms [77,78]. Like most repetitive DNA elements, ECVs are subjected to DNA methylation and histone modifications (i.e., H3K9me2), which are hallmarks of transcriptionally silent heterochromatin environments [79]. Furthermore, in silico mining of small RNA (sRNA) datasets and actual sequencing experiments have shown that a heterogenous population of 18–30 nt sRNAs that are homologous to ECVs are present at variable densities in a large range of angiosperms [22,31,68,69]. This pool of sRNAs is probably composed of subcategories acting in different silencing pathways, including transcriptional gene silencing (TGS) and post-transcriptional gene silencing (PTGS). For instance, the predominant function of plant 24-nt sRNAs is to guide the canonical RNA-directed DNA methylation (RdDM) pathway, while 21–22 nt sRNAs are usually involved either in non-canonical RdDM pathway or in PTGS [80,81,82].

Upon viral infection, plants produce a diversity of virus-derived sRNA as a defense mechanism to restrict viral replication and limit its spread (reviewed in [83,84]). In plants, Jakowitsch et al. [9] hypothesized that tobacco-endogenous pararetroviruses (TEPRVs) contribute to resistance against the cognate exogenous viruses, and Mette et al. [64] provided indirect experimental evidence supporting this hypothesis. Furthermore, it was demonstrated in insects that gene silencing targeting a non-retroviral EVE results in the inhibition of the replication of a closely related exogenous virus via gene silencing mechanisms [63]. Therefore, sRNA produced from ECVs could also play a central role in the defense against cognate viruses [85,86].

Recently, Valli et al. [87] reported the presence of a subset of ECVs with high 22-nt sRNA mapping density in the *Solanum lycopersicum* genome. These regions, called TSAs (transcriptionally competent siRNA areas), correspond to rearranged ECVs with inverted repeat (IR) conformations. TSAs can thus produce transcripts that are prone to the formation of hairpin structures known to promote the production of 22-nt siRNA by dicer-like protein 2 (DCL2) [87]. A similar situation was reported from the genome of soybean, in which inverted repeats derived from endogenous cucumber mosaic virus sequences (family *Bromoviridae*) have the potential to form a hairpin structure promoting the production of sRNAs [88]. Another study showed that caulimovirid- and TE-derived IR in soybean fuel the production of DCL2-dependant 22-nt sRNA [89]. Furthermore, in their analysis of sRNA landscapes in interspecific *Solanum* hybrids, Lopez-Gomollon et al. [90] found a predominant deregulation in loci corresponding to ECVs, affecting mainly the levels of 22-nt sRNA as compared to 21-nt and 24-nt species. Remarkably, Valli et al. further demonstrated that TSA-derived 22-nt sRNA can trigger the degradation of transcripts carrying the complementary sequence through PTGS [87]. Altogether, these results suggest that 22-nt sRNA produced from TSAs, and more generally from EVE-derived IRs, could play a central role in the defense against EVE amplification and/or against infection by cognate viruses through silencing by non-canonical RdDM or through PTGS [91].

## 4. Impact of ECVs on the Structure and Functions of Plant Genomes

The impact of ERVs on the structure and functions of vertebrate genomes is well documented [66], encompassing gene disruption [92], domestication of retroviral genes [93], and *cis*-acting regulatory elements such as promoters [94,95,96]. On the contrary, little is known about the impact of EVEs on the structure and functions of plant genomes, and the majority of data available concerns ECVs.

Some ECVs are found near or within host plant genes [22,52]. For example, 9% of the florendoviral EVEs of *Vitis vinifera* overlap with host genes, and 99% of them are located within introns [22]. However, there is still no evidence that these florendovirus insertions disrupt important genes, nor that florendovirus promoters are used to drive the expression of host plant genes. A petuvirus EVE was found inserted in a QTL associated with resistance against citrus tristeza virus in the genome of *Poncirus trifoliata* [52]. The AT repeat regions in which insertions were found could also be associated with host genes [51].

There is currently little evidence of the domestication by host plants of caulimovirid genes or regulatory elements. In 2019, Carrasco et al. [97] highlighted the importance of the AtMP gene in *Arabidopsis thaliana*, which consists of the fusion of the At1g37113 gene with a caulimovirid-derived MP gene. The downregulation of AtMP results in a small delay in plant development increases the negative effect of salinity on the efficacy of germination, and increases the resistance to an RNA virus. More recently, Serfraz et al. identified the presence of a badnavirus EVE in an *R1* resistance gene in the genome of brinjal eggplant (*S. melongena*) [66]. The *R1* gene family is involved in resistance against *Phytophthora infestans* in eggplant [98]. The insertion causes the disruption of an *R1* gene and results in the production of an alternative transcript (4–5 kb) via a putative alternative viral promoter (P2), but its evolutionary significance and biological outcomes are unknown.

Evidence of the impact of ECVs on plant biology remains limited. Only the work on the alternative badnavirus promoter in *S. melongena* and the work on caulimovirid MP homologs point in the direction of domestication of ECVs. However, these hypotheses will require further research in the future.

## 5. Conclusions

Almost 25 years after their discovery, ECVs have proved instrumental for our understanding of the long-term evolution of the *Caulimoviridae* and the dynamics of plant genomes through horizontal gene transfer. However, we still have only a very fragmented picture of their contribution to plant genomes and their diversity and virtually no indication of their impact on plant biology. Despite their ubiquity and abundance, ECVs remain largely neglected features during plant genome annotation because of a lack of dedicated tools for their proper annotation, hampering the development of paleovirology approaches. CAULIFINDER, a bioinformatics tool, was recently developed for the automated detection and annotation of ECVs in plant genomes [99], jointly with databanks that can be used to help with the classification of repetitive elements produced by standard repeat annotation pipelines. Use of CAULIFINDER should improve ECV annotation in plant genomes, foster comparative analyses across caulimovirid genera and their hosts, and characterize candidate functional ECVs. The search for ECVs has also been hampered by the taxonomic bias in plant genome assemblies, which have been largely focused on flowering plants. Here again, the recent release of genome sequences from modern representatives of ancient plant divisions brings promise for broader paleovirology studies. The increasing quality of plant genome assemblies will provide broader access to ECVs since many of these are found in highly repetitive regions such as hotspots, pericentromeres, and centromeres that are easier to assemble from high-quality sequencing data. These technical advances will help investigate the genomic distribution of ECVs, and their contribution to plant genomes, including the capture of beneficial genes and regulatory elements, and further investigate the long-term evolution and the origins of the *Caulimoviridae*.

## Figures and Tables

**Table 1:** Percent amino acid identities for fern and gymnosperm ECVs in RT amino acids sequences. Percent amino acid identities obtained using Blastp are shown. Those above cutoff value (55%) are in bold and highlighted in yellow. The name of putative viral species and genera is in bold. Complete names of putative viral species are: Gymnendovirus_1_Pabies: Gymnendovirus 1 Picea abies; Gymnendovirus_2_Gbilo: Gymnendovirus 2 Ginkgo biloba; Gymnendovirus_3_Pabies: Gymnendovirus 3 Picea abies; Gymnendovirus_4_Pabies: Gymnendovirus 4 Picea abies; PtaeV_1: Pinus taeda virus 1; PtaeV_2: Pinus taeda virus 2; PglaV_1: Pinus glauca virus 1; PglaV_2: Pinus glauca virus 2; GbilV: Gingko biloba virus.

	Gong & Han [24]	α-GECV PtaeV_2	β-GECV PglaV_2	δ-GECV PglaV_1	ε-GECV PtaeV_1	Ɣ-GECV GbilV
Diop et al. [11]						
**Gymnendovirus 1** Gymnendovirus_1_Pabies		44.1%	**73.0%**	50.2%	46.3	50.6%
**Gymnendovirus 2** Gymnendovirus_2_Gbilo		51.0%	52.0%	47.1%	48.0%	**56%**
**Gymnendovirus 3** Gymnendovirus_3_Pabies		43.7%	50.6%	**76.1%**	**58.6%**	48.2%
**Gymnendovirus 4** Gymnendovirus_4_Pabies		46.2%	53.7%	**57.7%**	**60.7%**	45.1%

## Data Availability

No new data were created or analyzed in this study. Data sharing is not applicable to this article.
